# The long non-coding RNA MSTRG.32189-PcmiR399b-*PcUBC24* module regulates phosphate accumulation and disease resistance to *Botryosphaeria dothidea* in pear

**DOI:** 10.1093/hr/uhae359

**Published:** 2025-01-03

**Authors:** Yuekun Yang, Shamei Lv, Xiaosan Huang, Ying He, Xiaoyan Zhang, Yu Liu, Guoping Wang, Ni Hong, Liping Wang

**Affiliations:** College of Plant Science and Technology, Huazhong Agricultural University, Wuhan 430070, China; Key Laboratory of Plant Pathology of Hubei Province, Huazhong Agricultural University, Wuhan 430070, China; Hebei Academy of Agriculture and Forestry Sciences, Shijiazhuang Fruit Tree Research Institute, Shijiazhuang 050061, China; College of Plant Science and Technology, Huazhong Agricultural University, Wuhan 430070, China; Key Laboratory of Plant Pathology of Hubei Province, Huazhong Agricultural University, Wuhan 430070, China; State Key Laboratory of Crop Genetics and Germplasm Enhancement, Centre of Pear Engineering Technology Research, Nanjing Agricultural University, Nanjing 210095, China; College of Plant Science and Technology, Huazhong Agricultural University, Wuhan 430070, China; Key Laboratory of Plant Pathology of Hubei Province, Huazhong Agricultural University, Wuhan 430070, China; College of Plant Science and Technology, Huazhong Agricultural University, Wuhan 430070, China; Key Laboratory of Plant Pathology of Hubei Province, Huazhong Agricultural University, Wuhan 430070, China; College of Plant Science and Technology, Huazhong Agricultural University, Wuhan 430070, China; Key Laboratory of Plant Pathology of Hubei Province, Huazhong Agricultural University, Wuhan 430070, China; College of Plant Science and Technology, Huazhong Agricultural University, Wuhan 430070, China; Key Laboratory of Plant Pathology of Hubei Province, Huazhong Agricultural University, Wuhan 430070, China; College of Plant Science and Technology, Huazhong Agricultural University, Wuhan 430070, China; Key Laboratory of Plant Pathology of Hubei Province, Huazhong Agricultural University, Wuhan 430070, China; College of Plant Science and Technology, Huazhong Agricultural University, Wuhan 430070, China; Key Laboratory of Plant Pathology of Hubei Province, Huazhong Agricultural University, Wuhan 430070, China

## Abstract

Pear ring rot disease (*Botryosphaeria dothidea*) is a significant threat to the healthy development of the pear industry. Recent research has identified the functional role of long non-coding RNAs (lncRNAs) in various biological processes of plants. The role of lncRNAs in the pear defense response remains unknown. In this study, transcriptome sequencing was used to analyze lncRNAs in pear stem infected with *B. dothidea*. It identified 3555 lncRNAs, of which 286 were significantly differentially expressed. GO and KEGG analyses showed that *cis*- and *trans*-regulated target genes were enriched in multiple disease resistance-related pathways. More specifically, MSTRG.32189, predicted as an endogenous target mimic (eTM), was significantly down-regulated in response to *B. dothidea* infection, and was confirmed to inhibit the cleavage effect of PcmiR399b on *PcUBC24.* OE-MSTRG.32189 transgenic *Arabidopsis* exhibited lower Pi content and weaker disease resistance to *Botrytis cinerea* compared with wild type. In pear callus, overexpression of MSTRG.32189 negatively regulated PcmiR399b, which decreased Pi content and reduced disease resistance. Overexpressing PcmiR399b in pear callus exhibited the opposite effects compared with OE-MSTRG.32189. Overexpression and knockout of *PcUBC24* further clarified that *PcUBC24* negatively regulates Pi content and disease resistance to *B. dothidea* infection. Furthermore, the ROS levels and expressions of disease resistance pathway-related genes were regulated by the MSTRG.32189-PcmiR399b-*PcUBC24* module in transgenic pear callus, which contributed to disease resistance. Overall, our results demonstrated the role of lncRNAs in the pear defense response, revealing that the MSTRG.32189-PcmiR399b-*PcUBC24* module regulates phosphate accumulation and disease resistance to *B. dothidea* infection in pear.

## Introduction

Long non-coding RNA (lncRNA) >200 nucleotides in length exists widely in animals, plants, and microorganisms [1]. lncRNAs were initially believed to be ‘dark matter’ without functions [2] and were recently revealed to play an extensive regulatory role in various biological processes, including biotic and abiotic stress responses in plants [3, 4]. lncRNAs were mainly categorized into four types based on their position in the genome, including long intergenic ncRNAs (lincRNAs), intronic ncRNAs (incRNAs), long non-coding natural antisense transcripts (lncNATs), and sense lncRNAs [5]. The function and regulation mechanism of lncRNAs with no protein encoding capability is emerging to be a fascinating area of research in plants. Although increasing numbers of lncRNAs were identified in different plant species with the development of bioinformatics and sequencing techniques, the functions of only a few lncRNAs were experimentally verified [4].

lncRNAs were reported to play regulatory roles in both plant development and stress responses [3]. In pear, *HILinc1* was reported to promote PbHSFA1b activity and enhance heat resistance [6]. As regulatory factors with strong spatiotemporal expression specificity, lncRNAs play a significant role in plant defense responses and partly determine the differences between resistant and susceptible varieties [7]. In tomato, lncRNA16397 was identified and revealed to regulate ROS accumulation and resistance to *Phytophthora infestans* by inducing expression of *SIGRX* [8]*.* In cotton, many resistance-related lncRNAs were identified and shown to be species-specific. Virus-induced silencing of GhlncNAT-*ANX2* and GhlncNAT-*RLP7* could increase resistance to *Verticillium dahlia* in cotton seedlings [9]. Transcriptome sequencing of three cultivars of Chinese cabbage showed that there were more differentially expressed lncRNAs in resistant lines than the susceptible line, and an lncNAT named MSTRG.19915 was verified to negatively regulate the resistance to downy mildew [10]. At present, most understanding of the roles of lncRNA in plant disease resistance is based on high-throughput sequencing identification and bioinformatics analysis. The specific function and mechanism of lncRNA in disease resistance need to be further investigated. Moreover, the existence of lncRNAs and their regulatory roles in the response to *Botryosphaeria dothidea* infection in pear need to be further explored.

Some of the lncRNAs could competitively bind miRNA to hinder its cleavage and repression of target mRNA expression. The lncRNAs of this kind were named endogenous target mimics (eTMs) or miRNA sponges [11]. eTMs were highly complementary to target miRNAs, with a nucleotide bulge at the middle of the matching sequence [12], and function by directly binding miRNA and positively regulating the corresponding target gene. Recent research has shown the regulatory roles of eTMs in horticultural plant growth and development [13, 14]. In lily, the eTM of miR171 was identified and found to boost somatic embryogenesis by preventing the cleavage of *lpSCL6* [13]*.* In citrus, researchers identified an lincRNA that has its highest expression level in fruit which could function as an eTM of csi-miR166c [14]. In addition, eTM was found to regulate disease resistance in plants. lncRNA23468 acts as an eTM to regulate the expression of *NBS-LRR* genes and disease resistance by absorbing miR482b in tomato [15]. It is also reported that lncRNA47980 modulates the resistance of tomato to *P. infestans* by regulating the contents of ROS and phytohormones [16]. There is a need for further investigation of whether eTMs could regulate disease resistance in pear.

Pear belongs to the Rosaceae family and is an economically important temperate fruit tree. There is a wide range of varieties of pear and they are widely cultivated worldwide, covering an area of ~1.3 million hectares [17]. Disease infections leading to low quality and export restrictions of pear hamper the healthy development of the industry and its economic benefits. Pear ring rot disease caused by *B. dothidea* is a difficult bottleneck for the pear industry. *Botryosphaeria dothidea* mainly infected branches, trunks, and fruits of pear trees and caused stem cankers, fruits rots, and even recession of tree growth [18]. Exploration and exploitation of resistance genes for pear breeding is a crucial solution for this problem. lncRNA, as an increasingly important regulator, has not yet been reported to be involved in defense responses in pear. In this study, 3555 lncRNAs were identified and a series of lncRNAs involved in the defense response in *cis*- or *trans*-regulating ways were screened based on transcriptome sequencing of *B. dothidea* infection in pear. Furthermore, we identified a significantly down-regulated eTM, MSTRG.32189, and explored its function in disease resistance and Pi homeostasis in pear by regulating the miR399b-*PcUBC24* module. It is of great value to conduct in-depth exploration and research on lncRNAs for future genetic improvement of resistant pear varieties.

## Results

### Identification and characterization of lncRNAs in pear

**Figure 1:**
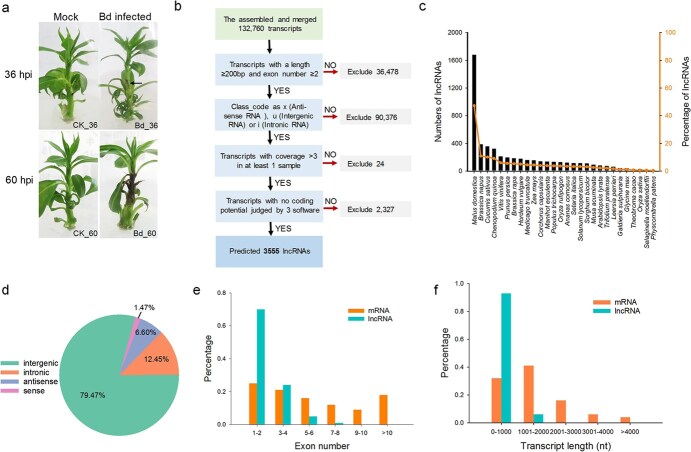
Identification and characterization of lncRNAs in pear stem infected with *B. dothidea* or mock-inoculated with H_2_O. **a** Symptoms of ‘Conference’ plantlets infected with *B. dothidea* for sequencing. **b** Bioinformatics pipeline for the systematic identification of lncRNAs in stem of ‘Conference’. **c** Conservation analysis of the identified lncRNAs with 28 other species. Black bars indicate the number of lncRNAs in pear homologous to those in other species; the orange line indicates the percentage of lncRNAs in pear homologous to those in other species. **d** Classification of the identified lncRNAs based on their genomic positions with protein-coding genes. **e**, **f** Distribution of exon number (**e**) and transcript length (**f**) of lncRNAs and mRNAs.

To explore the lncRNAs involved in the response of pear to *B. dothidea* infection, transcriptome sequencing was performed on *Pyrus communis* L. cv ‘Conference’ infected with *B. dothidea* and mock-inoculated with ddH_2_O at 36 and 60 hours post inoculation (hpi) (Fig. 1a). Each treatment contained three biological replicates and finally 12 libraries of data were obtained. The Q30 base percentage of all libraries was >93%, confirming the credibility of the sequencing data (Supplementary Data Table S1). After filtering, >60 million clean reads were generated from each sample and >88% of the reads could be uniquely mapped to the pear genome (Supplementary Data Table S2). Pearson correlation analysis indicated a strong correlation, with Pearson correlation coefficients >0.8 between the three biological replicates (Supplementary Data Fig. S1).

Based on the molecular characteristics of lncRNA, 3555 lncRNAs were finally identified from the total of 132 760 assembled transcripts by four-step screening pipeline analysis (Fig. 1b, Supplementary Data Fig. S2). The conservation of the identified lncRNAs in pear with other species was determined using BLAST software (E value <1e−5). Among the 28 randomly selected species, 1679 lncRNAs account for the highest percentage, with 47.2% homologous to those of *Malus domestica*, next to 10.89% homology to that of *Brassica napus*. The homology percentage of the other 20 species were <5% with those of pear, indicating the poor conservation existing between pear and other species (Fig. 1c). To further characterize the lncRNAs identified in pear stem, the expression level, exon number, and transcript length of lncRNAs were compared with those of mRNAs. The overall expression level of lncRNAs was obviously lower than that of mRNAs (Supplementary Data Fig. S3). The lncRNAs with one or two exons accounted for ~70%, while only 25% of the mRNAs had one or two exons. Furthermore, the transcript length of most lncRNAs was <1000 nucleotides, while ~68% of the mRNAs had >1000 nucleotides (Fig. 1e and f). This indicated that the identified lncRNAs in pear exhibit relatively lower expression levels and shorter lengths than mRNAs.

The identified lncRNAs, together with known lncRNAs annotated on the pear genome, were classified into different categories according to their genomic locations (Fig. 1d). Most of the lncRNAs were categorized as lincRNAs (79.47%), which showed intergenic locations, followed by incRNAs (12.45%) and lncNATs (6.6%), which were located in the intronic regions and the antisense strand of protein-coding genes, respectively (Fig. 1d). All identified lncRNA genomic locations and their putative classification are shown in Supplementary Data Table S3. Based on the location of the identified lncRNAs in the pear genome, we sifted out 650 lncNATs (Supplementary Data Table S4). KEGG enrichment analysis of target genes of lncNATs showed that the largest number of genes were involved in the ‘Plant–pathogen interaction’ pathway. In addition, many genes were involved in other disease resistance-related pathway, such as ‘Autophagy’ and ‘Cutin, suberine and wax biosynthesis’ (Supplementary Data Fig. S4a). lncNATs have been reported to negatively regulate the overlapping protein-coding genes [10]. We performed co-expression analysis of lncNAT-mRNA gene pairs to investigate the regulation relationship between the lncNAT and target protein-coding genes. After filtering, four protein-coding genes were identified to be negatively regulated by corresponding lncNATs (Supplementary Data Fig. S4b and c). The gene *Cytochrome P450 94A1* (*CYP94A1*) in the lncNAT–mRNA pair with the highest correlation was reported to be involved in cutin biosynthesis and plant defense [19].

### Prediction and characterization of c*i*s- and *trans*-regulated lncRNA–mRNA gene pairs in response to *B. dothidea* infection

**Figure 2:**
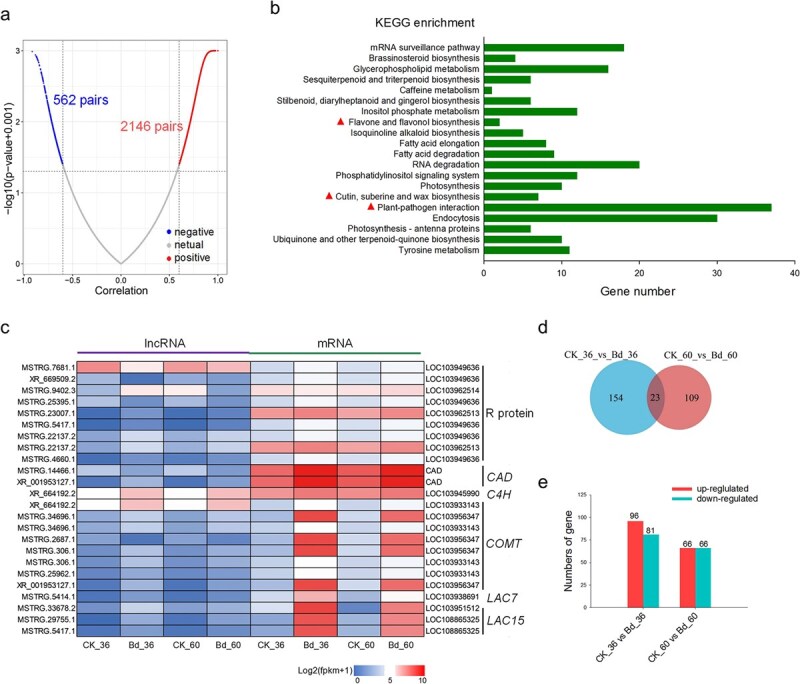
Characterization of *cis*- or *trans*-regulated lncRNA-mRNA gene pairs and numbers of differentially expressed lncRNAs in response to *B. dothidea* infection. **a** Co-expression correlation analysis of predicted *cis*-regulated lncRNA–mRNA pairs. **b** KEGG enrichment of *cis*-target genes correlated with neighboring lncRNAs; disease resistance-related items are highlighted with red triangles. **c** Heat map analysis of R genes and lignin biosynthesis genes *trans*-regulated by differentially expressed lncRNAs. **d** Venn diagram analysis of differentially expressed lncRNAs. **e** Numbers of lncRNAs with up- and down-regulated expression.

It has been reported that lncRNAs function through *cis*- and *trans*-regulating protein-coding genes [3]. By searching the nearby genes in the genome, 2466 identified lncRNAs were predicted to be *cis*-functional lncRNAs (Supplementary Data Table S5, Fig. 2). Co-expression analysis was performed on neighboring lncRNA–mRNA pairs to further explore the effects of *cis*-functional lncRNAs. The results showed that 2146 pairs have a positive correlation while 562 pairs exhibit a negative correlation, indicating lncRNAs can *cis*-regulate neighboring genes in a predominantly positive way (Fig. 2a). KEGG enrichment analysis showed that the correlated nearby genes were enriched in several disease resistance-related pathways, including ‘Plant–pathogen interaction’, ‘Cutin, suberine and wax biosynthesis’, and ‘Flavone and flavonol biosynthesis’ (Fig. 2b).

Based on the expression correlation analysis, 995 identified lncRNAs were predicted to be *trans*-functional lncRNAs (Supplementary Data Table S6). We characterized the functions of *trans*-regulating lncRNAs by Gene Ontology (GO) enrichment analysis of target genes (Supplementary Data Fig. S5). R genes and lignin biosynthesis pathway gene-mediated disease resistance is conserved in plants [20]. We selected differentially expressed lncRNAs that were predicted to *trans*-regulate R genes or lignin biosynthesis pathway genes and present the expression levels as a heat map. Among the nine screened regulatory pairs of lncRNA–R genes, five pairs were positively correlated genes, including MSTRG.23007.1–LOC103962513, and four pairs were negatively correlated genes, including MSTRG.7681.1–LOC103949636. In addition, we found 15 lncRNA–mRNA gene pairs involved in the lignin biosynthesis pathway, such as MSTRG.34696.1–*PcCOMT* and MSTRG.5414.1–*PcLAC7* (Fig. 2c). The expression level of two pairs of predicted *cis*-regulated lncRNA–mRNAs and two pairs of predicted *trans*-regulated lncRNA–mRNAs were verified by semiquantitative RT–PCR and the expression patterns were consistent with the FPKM values of RNA-seq (Supplementary Data Fig. S6). Based on the above results, it can be hypothesized that differentially expressed lncRNAs can regulate the expression of disease resistance-related genes in a *cis* or *trans* way.

### Expression pattern and functional analysis of differentially expressed lncRNAs in pear response to *B. dothidea* infection

To explore the potential lncRNAs participating in disease resistance, the expression pattern of lncRNAs was analyzed and the differentially expressed lncRNAs were screened. The expression levels of lncRNAs in samples inoculated with *B. dothidea* were compared with those of the samples inoculated with ddH_2_O at 36 and 60 hpi. There was a total of 286 differentially expressed lncRNAs at 36 and 60 hpi, while only 23 lncRNAs were differentially expressed at both 36 and 60 hpi (Fig. 2d, Supplementary Data Table S7).

**Figure 3:**
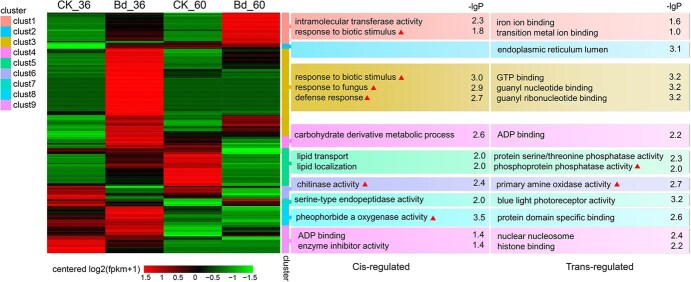
Clustering analysis and target gene GO enrichment of differentially expressed lncRNAs. The differentially expressed lncRNAs were divided into nine clusters based on the expression pattern and the top one to three significant GO items based on –lgP are shown on the right (disease resistance-related items are highlighted with red triangles)

Based on the expression patterns for different treatments and inoculation time points, we divided the differentially expressed lncRNAs into nine clusters (Fig. 3, Supplementary Data Table S8). Then, GO analysis of the target genes was performed to understand the potential function of lncRNAs in each cluster (Fig. 3). Cluster 3 contained the largest number of lncRNAs and was significantly up-regulated compared with the control at the early stage (36 hpi) of pathogen infection. Remarkably, the predicted *cis*-regulated lncRNA genes, such as *phytohormone-binding protein* and *defensin-like protein 2*, targeted with MSTRG.33361.1 and XR_666716.2 in cluster 3, were mostly involved in the biotic stress-related GO items, indicating that this cluster of lncRNAs was responsive to pathogen infection and plays an important role in regulating disease resistance. In addition, *cis*- or *trans*-target genes in clusters 1, 5, 6, and 8 were also significantly involved in disease resistance-related pathways (Fig. 3).

### MSTRG.32189 expressed in pear stem tissue could function as an endogenous target mimic of miR399b to inhibit the cleavage effect on *PcUBC24*

eTMs are lncRNAs of a kind that could bind miRNA and hinder its cleavage activity to mRNA [12]. Among the newly identified lncRNAs in pear, three potential eTMs, MSTRG.15284.5, MSTRG.10113.1, and MSTRG.32189, were identified for binding miR319b, miR482d, and miR399b, respectively. MSTRG.10113.1 binds to miR482d with a two-nucleotide bulge between the 9th and 10th bases. MSTRG15284.5–miR319b and MSTRG.32189–miR399b binding pairs exhibit a three-nucleotide bulge between the 10th and 11th bases (Supplementary Data Fig. S7).

**Figure 4:**
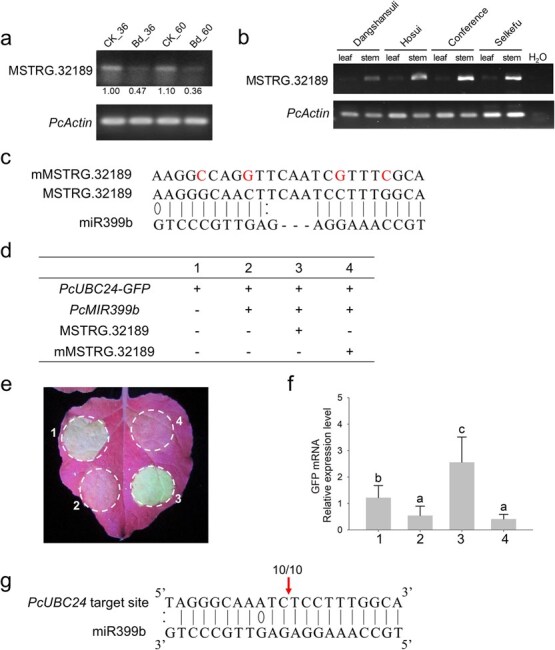
MSTRG.32189 expression level analysis and verification of the effect of MSTRG.32189 on miR399b-guided cleavage activity on *PcUBC24* with transient expression assays in tobacco. **a** Expression profile verification of MSTRG.32189 in response to *B. dothidea* infection by semiquantitative RT–PCR. **b** Expression profiles of MSTRG.32189 in leaf and stem tissues of four pear species, including *Pyrus bretschneideri* cv. ‘Dangshansuli’, *P. pyrifolia* cv. ‘Fengshui’, *P. communis* cv. ‘Conference’, and *P. sinkiangensis* cv. ‘Selkefu’, by semiquantitative RT–PCR. **c** Mutant site of mMSTRG.32189 (marked with red font) at predicted matching sequence. **d** Different combinations of constructs infiltrating tobacco leaves at different positions in tobacco leaves. **e** Expression level of *PcUBC24*-*GFP* for different combinations photographed under UV light. **f** Relative expression level of *GFP* mRNAs from different infiltrating sites examined by RT–qPCR. **g** Cleavage site verification of *PcUBC24* at position 2 infiltrated with 35S:*MIR399b* and 35S:*PcUBC24*-*GFP* by 5′-RLM-RACE. Red arrow indicates the inferred cleavage site, and the numbers above sequences indicate clones of detected cleavage site relative to total clones.

Among the three predicted eTMs, MSTRG.32189 was the only significantly differentially expressed lncRNA and was down-regulated after *B. dothidea* infection, at both 36 and 60 hpi, compared with the mock control (Supplementary Data Table S7). Semiquantitative RT–PCR results showed that the expression level of MSTRG.32189 was significantly down-regulated compared with the control sample after infection with *B. dothidea* at 36 and 60 hpi (Fig. 4a), which was consistent with the high-throughput sequencing results (Supplementary Data Table S7). In addition, we also analyzed the expression patterns of MSTRG.32189 in response to *Diaporthe eres* and *Valsa pyri*, which mainly infect the trunk and branches of pear, and the results showed that the expression levels of MSTRG.32189 were also down-regulated (Supplementary Data Fig. S8). We therefore deduced that MSTRG.32189 might play a role in the disease resistance response and focused on it for further investigation.

Semiquantitative RT–PCR was performed to investigate the expression level of MSTRG.32189 in leaf and stem tissues of pear plantlets. To ensure representativeness, we randomly selected eight cultivars from five major cultivated species and one rootstock of pear: *Pyrus bretschneideri* ‘Dangshansuli’ and ‘Huangguan’, *P. pyrifolia* ‘Hosui’, *P. communis* ‘Conference’, *P. sinkiangensis* ‘Selkefu’ and ‘Korla’, *P. ussuriensis* cv. ‘Jingbai’, and the rootstock ‘Qingzhen’. The results showed that MSTRG.32189 exhibits higher expression in stem tissues of four pear species, while it has lower expression levels in leaf tissues, with observation of no apparent band or a weak band in electrophoretic semiquantitative RT–PCR products (Fig. 4b, Supplementary Data Fig. S8c).

To test the effect of MSTRG.32189 on miR399b-guided cleavage activity, we conducted a transient expression assay in tobacco. *PcUBC24* was predicted as the target gene of PcmiR399b in previous research about small RNA sequencing of pear shoots [21]. We made constructs expressing green fluorescent protein (GFP) fused with the target site of the *PcUBC24* gene at the 5′-terminus (*PcUBC24-GFP*). PcmiR399b, MSTRG.32189, and mMSTRG.32189 (four bases were mutated at the predicted binding site shown in Fig. 4c) were cloned into pBI121 to generate 35S:*MIR399b*, 35S:MSTRG.32189, and 35S:mMSTRG.32189 constructs. The four construct combinations used in co-expression experiments are shown in Fig. 4d.

Compared with the position expressing *PcUBC24*-*GFP* singly, GFP fluorescence intensity decreased obviously at the position co-expressing PcmiR399b and *PcUBC24*-*GFP* (Fig. 4e). The cleavage of *PcUBC24* by PcmiR399b was verified by 5′-RLM-RACE and the sequencing results showed that the cleavage site was at the 10th and 11th nucleotides of the predicted matching sequence (Fig. 4g). However, the GFP intensity recovered to a high level when co-expression of MSTRG.32189 with PcmiR399b and *PcUBC24*-*GFP* was performed, indicating that the cleavage effect of miR399b on *PcUBC24*-*GFP* was suppressed by the expression of MSTRG.32189. Additionally, co-expressing the mutated MSTRG.32189 (mMSTRG.32189), which contains four-base mutations within the matching sequence, lost the suppression effect of miR399b cleavage activity, indicating that MSTRG.32189 functions via pairing with miR399b at the predicted site (Fig. 4c and e). The *GFP* mRNA levels were further confirmed by RT–qPCR, which was consistent with the fluorescence intensity (Fig. 4f). The above results demonstrate that MSTRG.32189 could act as an eTM to inhibit the cleavage of *PcUBC24* by PcmiR399b.

### MSTRG.32189 negatively regulated disease resistance to *Botrytis cinerea* infection in transgenic *Arabidopsis*

**Figure 5:**
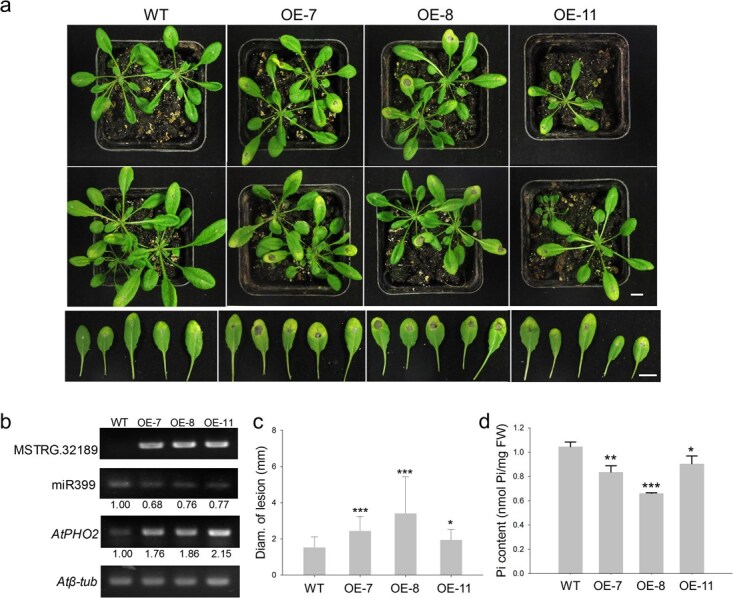
Expression level analysis of MSTRG.32189 and determination of resistance in OE-MSTRG.32189 *Arabidopsis* to *Botrytis cinerea* infection. **a** Disease phenotype of whole plants and representative leaves at 3 days post-inoculation with *B. cinerea* spores. Scale bar = 1 cm. **b** Expression levels of miR399b and *PHO2* in OE-MSTRG.32189 transgenic *Arabidopsis* determined by semiquantitative RT–PCR. **c** Diameter of lesions in WT and OE-MSTRG.32189 lines. **d** Free Pi contents. Error bars indicate mean ± standard deviation from biological replicates. Asterisks indicate significant differences from WT (*t*-test): ^*^P < 0.05; ^**^P < 0.01, ^***^P < 0.001.

To verify the function of MSTRG.32189 in disease resistance, we firstly transformed MSTRG.32189 into wild-type (WT) *Arabidopsis* seedlings and obtained three overexpressing lines, designated OE-7, OE-8, and OE-11 (Fig. 5). The growth phenotype of OE-MSTRG.32189 lines at 4 weeks post-germination showed no difference compared with WT seedlings, which indicated that overexpressing MSTRG.32189 did not affect the growth of *Arabidopsis* (Supplementary Data Fig. S9). Semiquantitative RT–PCR was performed to confirm the regulation by MSTRG.32189 of miR399b and corresponding target gene *AtPHO2* in *Arabidopsis* [22]. The results showed that in three transgenic lines with overexpression of MSTRG.32189, miR399b was obviously down-regulated and target gene *AtPHO2* significantly up-regulated compared with WT seedlings (Fig. 5b). The expression patterns of MSTRG.32189, miR399b, and *AtPHO2* indicated that MSTRG.32189 could function as an eTM of miR399b in *Arabidopsis* to regulate miR399b-*AtPHO2*. We cloned the CDS sequence of *PcUBC24*, the target gene of miR399b in pear, which can encode 916 amino acids (GenBank accession number PP104289). By analyzing the conserved domains of PcUBC24 in pear and AtPHO2 in *Arabidopsis*, it was found that both genes have conserved UBCc domains encoding E2 ubiquitin-conjugating enzymes and are homologous to each other (Supplementary Data Fig. S10).

The OE-MSTRG.32189 lines were further challenged with fungal pathogen *Botrytis cinerea.* The results showed that the OE-MSTRG.32189 lines exhibited more severe symptoms than WT after *B. cinerea* infection (Fig. 5a). The diameter of lesions in OE-MSTRG.32189 significantly increased by ~26–123% compared with WT (Fig. 5c). Based on the degradation activity of phosphate transporters of *PHO2*, free Pi content was determined in OE-MSTRG.32189 lines and WT seedlings. The results showed that the Pi content in OE-MSTRG.32189 lines was significantly reduced by 10– 34% compared with WT (Fig. 5d). The above results indicated that overexpression of MSTRG.32189 in *Arabidopsis* could reduce Pi accumulation and disease resistance to *B. cinerea*.

### MSTRG.32189 negatively regulates Pi accumulation and disease resistance to *B. dothidea* infection in transgenic pear callus

**Figure 6:**
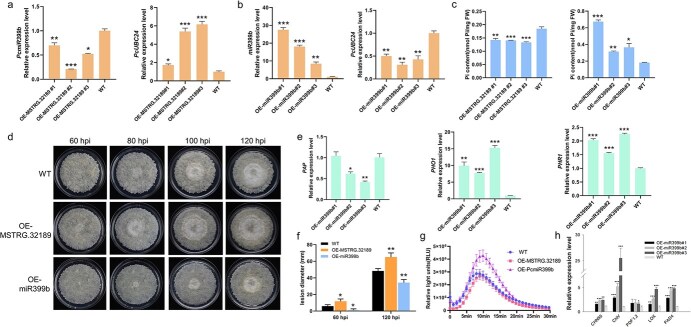
Functional validation of MSTRG.32189 and PcmiR399b in transgenic pear callus. **a** Expression level of miR399b and *PcUBC24* in WT and OE-MSTRG.32189 pear callus. **b** Expression level of miR399b and *PcUBC24* in WT and OE-PcmiR399b pear callus. **c** Pi contents of OE-MSTRG.32189 and OE-miR399b pear callus compared with WT. **d** Representative phenotypes of WT, OE-MSTRG.32189, and OE-miR399b pear callus infected with *B. dothidea* at different time points. **e** Relative expression level of phosphorus homeostasis-regulated genes in OE-miR399b and WT pear callus. **f** Lesion diameters in WT, OE-MSTRG.32189, and OE-miR399b pear callus infected with *B. dothidea* at 60 and 120 hpi. **g** ROS production dynamics in WT, OE-MSTRG.32189, and OE-PcmiR399b callus after chitin treatments. **h** Expression of disease resistance pathway-related genes in OE-miR399b and WT pear callus. Error bars indicate mean ± standard deviation from biological replicates. Asterisks indicate significant differences from WT (*t*-test): ^*^P < 0.05; ^**^P < 0.01; ^***^P < 0.001.

To further investigate the function of MSTRG.32189 in pear, we used pear callus as material for a transgenic assay. A construct overexpressing MSTRG.32189 was introduced into pear callus by *Agrobacterium*-mediated transformation. Three independent positive lines for OE-MSTRG.32189 were obtained and detected by PCR using DNA as template (Supplementary Data Fig. S11a). RT–qPCR results showed that miR399b was significantly decreased by 25–75%, while the corresponding target gene *PcUBC24* was up-regulated compared with WT pear callus in OE-MSTRG.32189 callus (Supplementary Data Fig. S11, Fig.  6a). This further confirmed that MSTRG.32189 could function as an eTM to positively regulate *PcUBC24* by decoying PcmiR399b in pear.

Based on the function of E2 ubiquitin-conjugating enzyme in regulating phosphate accumulation, we determined the Pi content of the WT and transgenic callus. Compared with the WT, the Pi content was significantly reduced by 22–28% in OE-MSTRG.32189 (Fig. 6c).

WT callus and transgenic lines of OE-MSTRG.32189 were further inoculated with *B. dothidea*. The results showed that the expansion rate of *B. dothidea* infection was faster in OE-MSTRG.32189 callus tissue than WT, and the lesion diameter increased by 34% compared with WT at 120 hpi (Fig. 6d and f). ROS is a key signal in the defense response [23]. We performed ROS determination to better understand how MSTRG.32189 regulates disease resistance. The dynamics of chitin-induced ROS levels in different transgenic calluses and WT callus were determined using a luminol-based assay [24]. ROS levels of different calluses peaked at ~10 min after treatment with chitin and the maximum values of OE-MSTRG.32189 were lower than in WT (Fig. 6g). The expression levels of genes related to disease resistance pathways were also measured in OE-MSTRG.32189 callus tissue and WT, and the results showed that MSTRG.32189 had effects on the expression of genes related to disease resistance pathways to some extent (Supplementary Data Fig. S12).

### PcmiR399b could positively regulate the resistance to *B. dothidea* infection and Pi accumulation in pear callus

For further investigation of whether MSTRG.32189 functions by regulating miR399b, we simultaneously constructed plasmids overexpressing PcmiR399b and transformed pear callus to obtain transgenic OE-PcmiR399b callus. Positive lines were detected by PCR using DNA as template (Supplementary Data Fig. S11b). In three OE-miR399b lines, RT–qPCR results further confirmed that miR399b was significantly up-regulated 7- to 28-fold and the expression level of *PcUBC24* was down-regulated to ~40% compared with the WT callus (Fig. 6b).

Compared with the WT, the Pi content was significantly increased by ~72–272% in OE-miR399b, which further suggests that PcmiR399b positively regulates phosphate accumulation (Fig. 6c). We also obtained transgenic pear callus silencing PcmiR399b using an STTM construct. The phosphate content in the three lines of STTM-miR399b decreased by 9–28% compared with the WT (Supplementary Data Fig. S13). To investigate whether the changes in phosphate content were due to an overall effect on phosphorus metabolism and transport, we examined the expression level of key phosphorus homeostasis-regulated genes by RT–qPCR. Purple acid phosphatases (PAPs) in plants release phosphorus from organic forms [25]. The RT–qPCR results showed that the expression of *PAP* was significantly down-regulated in OE-miR399b and up-regulated in STTM-miR399b. This revealed that changes in miR399b deficiency expression activated the release and utilization of phosphorus in transgenic lines. The expression levels of *PHOSPHATE1* (*PHO1*), a gene important for Pi transport, and *PHR1*, another gene related to Pi starvation response, were also examined in transgenic callus. The results showed that both genes were significantly down-regulated in the STTM-miR399b transgenic lines and up-regulated in the OE-miR399b lines, indicating that the uptake and transport capacity of Pi in the transgenic lines was positively correlated with the expression of PcmiR399b (Fig. 6e, Supplementary Data Fig.  S14).

The transgenic lines of OE-miR399b and WT callus were further inoculated with *B. dothidea* to detect differences in disease resistance. Through observation and statistical analysis at different time points, the lesion expansion rate of OE-miR399b was lower than that of WT and lesion diameter at 120 hpi in OE-miR399b callus was significantly reduced by ~30% compared with that of the WT callus (Fig. 6d and f). In addition, by measuring ROS levels, the results showed that the maximum value of ROS production in OE-miR399b was significantly increased by ~50% compared with WT callus (Fig. 6g). The expression of disease resistance signaling pathway genes such as *PDF1.2* and *LOX* in the JA pathway, and *CNH50* and *ChiV* in the SA signaling pathway were determined by RT–qPCR. The results showed that expressions were all significantly up-regulated in OE-miR399b relative to those of WT (Fig. 6h).

### *PcUBC24* negatively regulates resistance to *B. dothidea* infection and Pi accumulation in pear callus

MSTRG.32189 and PcmiR399b, as non-coding RNAs, were assumed to ultimately affect disease resistance by regulating the target protein-coding gene *PcUBC24*. Therefore, we transformed pear callus with overexpression and knockout constructs for *PcUBC24* to essentially investigate the functions of the MSTRG.32189-PcmiR399b-*PcUBC24* module.

**Figure 7:**
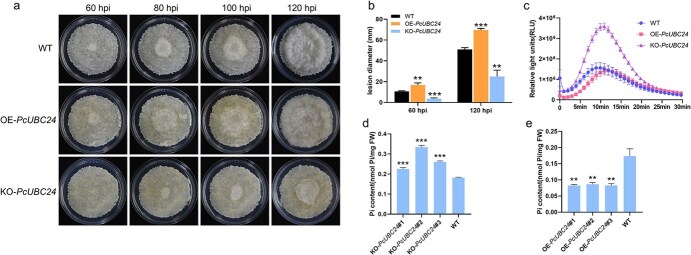
Functional validation of *PcUBC24* in transgenic pear callus. **a** Representative phenotypes of WT, OE-*PcUBC24*, and KO-*PcUBC24* pear callus infected with *B. dothidea* at different time points. **b** Lesion diameters in WT, OE-*PcUBC24*, and KO-*PcUBC24* pear callus infected with *B. dothidea* at 60 and 120 hpi. **c** ROS production dynamics in WT, OE-*PcUBC24*, and KO-*PcUBC24* callus after chitin treatments. **d**, **e** Pi content determination of KO-*PcUBC24* (**d**) and OE-*PcUBC24* (**e**) pear callus compared with WT. Error bars indicate mean ± standard deviation from biological replicates. Asterisks indicate significant differences from WT (*t*-test): **P < 0.01; ***P < 0.001.

After *Agrobacterium*-mediated transformation, pear callus transformed with overexpression and knockout constructs carrying the *GFP* coding sequence were detected to emit green fluorescence under UV light, whereas WT callus did not. Three transgenic lines of each construct were also identified by PCR using DNA as template (Supplementary Data Fig. S15). OE-*PcUBC24* transgenic callus was further confirmed by RT–qPCR to show a significant increase in *PcUBC24* expression of three lines relative to WT (Supplementary Data Fig. S16). In addition, the mutation efficiency in KO-*PcUBC24*-transformed pear callus using the CRISPR construct was assessed (Supplementary Data Fig. S17). The target gene fragments were amplified and 30 clones of each line were randomly selected for Sanger sequencing. The sequencing results showed that all clones had insertion or deletion mutations at the target position, which led to premature termination of the translation (Supplementary Data Fig. S18). The above results indicated that positive transgenic calluses of OE-*PcUBC24* and KO-*PcUBC24* were successfully obtained. Pi accumulation was detected in OE-*PcUBC24* and KO-*PcUBC24* pear callus in comparison with WT. The results demonstrated a 53% decline in free phosphate content of OE-*PcUBC24* callus Conversely, in KO-*PcUBC24* pear callus there was an increase ranging from 22% to 83% in free phosphate content compared with WT (Fig. 7d and e).

Subsequently, OE-*PcUBC24*, KO-*PcUBC24*, and WT calluses were inoculated with *B. dothidea* for disease resistance evaluation. The lesion expansion rate in OE-*PcUBC24* callus tissue was significantly faster than that in WT; its lesion diameter increased significantly by 35% compared with WT at 120 hpi. The expansion rate of lesions in KO-*PcUBC24* callus tissue was significantly slower than that in WT; its lesion diameter was significantly reduced by 50% relative to WT at 120 hpi (Fig. 7). Additionally, we transiently overexpressed the target gene *PcUBC24* in pear fruits and inoculated them with *B. dothidea* to observe disease development. Compared with fruits transformed with empty vector served as the control, lesion diameter increased significantly in OE-*PcUBC24* fruits (Supplementary Data Fig. S19). ROS determination was performed on OE-*PcUBC24*, KO-*PcUBC24* transgenic pear callus, and WT. The results showed that ROS production was significantly increased in KO-*PcUBC24* callus and its peak value was two times higher than that of WT, while, the peak value of ROS production in OE-*PcUBC24* callus was delayed and the maximum value was relatively lower than that in WT (Fig. 7c).

## Discussion

*Botryosphaeria dothidea* infection of branches, trunks, and fruit of pear trees causes pear ring rot disease and is detrimental to the healthy development of the pear industry [18]. Breeding for resistant pear varieties is the main solution for this problem. Research on the role of pear lncRNAs in regulating the defense mechanism to *B. dothidea* infection could provide candidate resistance genes for pear breeding. As important regulatory genes, lncRNAs were reported to be involved in multiple biological processes, including the defense response [7, 9]. This study screened a series of lncRNAs involved in pear defense against *B. dothidea* infection by bioinformatics analysis and demonstrated by experimental investigation that the newly identified MSTRG.32189 could function as an eTM of miR399b to regulate phosphate accumulation and disease resistance in pear.

Bioinformatics analysis firstly provides some insight into the molecular characteristics of lncRNA regulation involved in the pear response to *B. dothidea*. The lncRNAs identified in pear exhibited relatively lower expression levels and shorter lengths than mRNAs and showed extremely low conservation with other species, which was consistent with the traits of lncRNAs in previous reports [26, 27]. A total of 286 differentially expressed lncRNAs were screened in response to pathogen infestation, and the putative *cis*- and *trans*-regulated genes were enriched in a number of disease resistance-related pathways. For predicted *cis*-regulated lncRNA–mRNA gene pairs, we found that the regulation relationship operated mainly in a positive way and *cis*-correlated target genes were significantly enriched in several disease resistance-related pathways (Fig. 2a and b). As for *trans*-functional lncRNAs, a series of differentially expressed lncRNAs were found to be *trans*-correlated with R genes and lignin biosynthesis pathway genes (Fig. 2c), which had been proved to positively regulate disease resistance in plants [28]. In *Populus*, six modules responsive to salt stress were identified through co-expression networks involving 426 lncRNAs, of which 30 lncRNAs were found to have the potential to regulate salt stress-responsive homologous genes simultaneously through *cis*- and *trans*-regulation [27]. In Chinese cabbage, clustering and GO analysis screened a number of differentially expressed lncRNAs in disease-resistant varieties that specifically responded to pathogen infestation and were involved in the disease resistance pathway [10]. In this study, bioinformatics analysis helped us focus on a portion of the key lncRNAs, such as cluster 3, involved in disease resistance to *B. dothidea.* The expression levels of four *cis*- and *trans*-regulated lncRNA–mRNA gene pairs were verified by RT–qPCR, and this was consistent with the results of high-throughput sequencing analysis. This provided potential lncRNA gene resources for the genetic improvement of disease-resistant pear varieties.

To know about the identified lncRNAs more specifically, we screened three candidate eTMs through bioinformatics analysis (Supplementary Data Fig. S7). The selected MSTRG.32189 was significantly down-regulated after *B. dothidea* infection, and its function was further explored. Based on the genome mapping results, MSTRG.32189 was located in the intergenic region (Supplementary Data Table S3). The co-expression of MSTRG.32189, PcmiR399b, and *PcUBC24*-*GFP* in tobacco leaves confirmed the ability of MSTRG.32189 to decoy miR399b through the predicted matching sequence as an eTM and the cleavage ability of PcmiR399b on *PcUBC24* (Fig. 4). Our further investigation on the newly identified differentially expressed eTM, MSTRG.32189 in pear, aims to add new insights into the role of eTMs in fruit trees.

As a conserved miRNA in plants, miR399b targets genes encoding an E2 ubiquitin-conjugating enzyme which functions in the degradation of phosphate transporters and regulation of Pi homeostasis [22, 29, 30]. In this study, the newly identified MSTRG.32189 was verified to be able to down-regulate PcmiR399b and positively regulate the expression of the target gene *PcUBC24* (Fig. 6a), confirming that MSTRG.32189 could function as an eTM to regulate the PcmiR399b-*PcUBC24* module in pear. Our results of Pi determination in transgenic *Arabidopsis* and pear callus also clarified the negative regulatory effect of MSTRG.32189 on phosphate accumulation by regulating PcmiR399b-*PcUBC24* and phosphorus homeostasis-regulated genes (Figs 5f and 6c and e). Several previous research studies have shown that the miR399-*UBC24* module affects phosphate homeostasis by different mechanisms in plants [31]. In citrus, researchers demonstrated that the CsmiR399-*CsUBC24* module can participate in the regulation of male fertility and reproductive development [32]. In maize, two lncRNAs, *PILNCR2* and *PILNCR1*, are involved in regulating phosphorus uptake efficiency by different mechanisms [30, 33].

To further validate the effect of MSTRG.32189 on plant disease resistance, transgenic *Arabidopsis* and pear callus were challenged with *Botrytis cinerea* and *Botryosphaeria dothidea* infection, respectively. The results showed that MSTRG.32189 negatively affect disease resistance by regulating PcmiR399b-*PcUBC24* and suggest that changes in disease resistance are positively correlated with phosphate accumulation (Fig. 6). Previous research in tomato showed that eTMs could participate in regulating disease resistance in plants, revealing that lncRNA23468 and lncRNA39896 could function as an eTM to impair the cleavage effect of miR482 or miR166b, respectively, on NBS-LRR genes or *SlHDZ* and further contribute to disease resistance to *P. infestans* [15, 34]. In order to understand the possible reasons behind the impact of MSTRG.32189 on disease resistance, we further investigated the ROS levels in different transformants. The results showed that ROS levels were positively correlated with disease resistance as well as phosphate content in different transformants (Figs 6 and 7). This suggests that the MSTRG.32189-miR399b-*PcUBC24* module may regulate pear disease resistance by affecting the ROS signaling pathway. In addition, since MSTRG.32189 functions by regulating PcmiR399b, we further clarified that PcmiR399b can affect phosphorus homeostasis genes and disease resistance pathway-related genes in pear.

Nutritional imbalances due to excess or deficiency of nutrients may cause changes in plant disease resistance [35]. In citrus, endogenous phosphate levels were related to the degree of symptoms of huanglongbing (HLB), which develops more severely in the presence of phosphorus deficiency [36]. In previous studies, miR399 has been reported to participate in regulating plant disease resistance. In *Arabidopsis*, both phosphorus accumulation and overexpression of miR399 can enhance its resistance to necrotrophic and hemibiotrophic pathogens [37]. In rice, conversely, overexpressing miR399 with phosphate accumulation could enhance susceptibility to rice ragged stunt virus (RRSV) [38]. In this study, function validation experiments confirmed that down-regulation of MSTRG.32189 expression in response to *B. dothidea* infestation could enhance disease resistance by regulating PcmiR399b-*PcUBC24*. In addition, a positive correlation was found between phosphate content and ROS accumulation in different transgenic lines, which may be intrinsically due to cross-talk between signaling pathways. Through expression analysis, elevated ROS levels were confirmed to further activate disease resistance-related pathway genes and defense responses [23]. The results we obtained suggest that the effect of MSTRG.32189-PcmiR399b-*PcUBC24* on disease resistance may be due to a shift in defense-related hormonal signaling resulting from fluctuations in phosphorus nutrient content. Phosphorus is an important nutrient and the efficiency of its absorption and utilization by plants directly affects the growth and development of plants and phosphate fertilizer requirements [39]. Based on our results, genetic improvement of the MSTRG.32189-PcmiR399b-*PcUBC24* module is expected to achieve the dual role of enhancing phosphorus utilization efficiency and disease resistance in pear.

**Figure 8:**
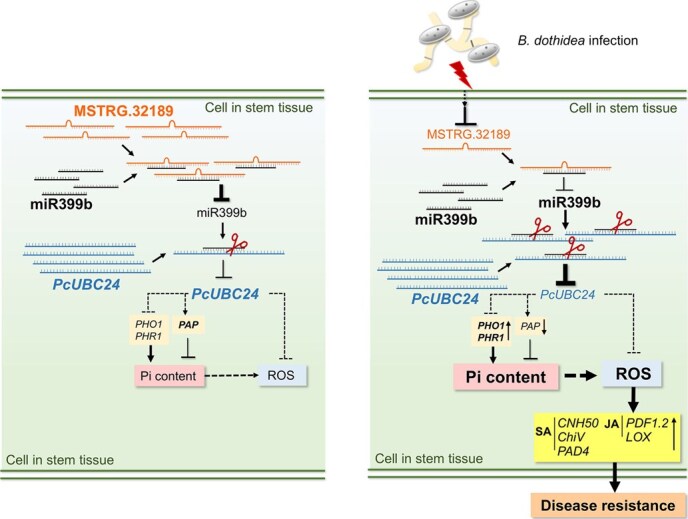
A proposed model of the regulatory role of the MSTRG.32189-PcmiR399b-*PcUBC24* module in pear resistance to *B. dothidea.* Expression of MSTRG.32189, as an eTM of PcmiR399b expressed in stem tissue, is inhibited during *B. dothidea* infection, thus enhancing cleavage of the target gene *PcUBC24* by PcmiR399b. Reduced *PcUBC24* expression could lead to attenuated degradation of phosphate transporter activity, which increases phosphate accumulation and activation of ROS signaling. The immune response is then activated to increase resistance to *B. dothidea.*

In conclusion, we have established a schematic diagram to explain how MSTRG.32189 regulates pear resistance to *B. dothidea* infection. Relative to the uninfected state, MSTRG.32189 was significantly down-regulated in response to *B. dothidea* infection. The reduction in expression of MSTRG.32189, which may act as an eTM of PcmiR399b, resulted in an increased cleavage effect on *PcUBC24* mRNA. Reduced *PcUBC24* expression may lead to attenuated degradation of phosphate transporter activity and changes in phosphorus homeostasis-regulating gene expression, which increases phosphate accumulation, activation of ROS and defense-related signaling in the response to *B. dothidea* relative to the uninfected state. The immune response is then activated to increase resistance to *B. dothidea* (Fig. 8).

## Materials and methods

### Plant materials and growth conditions

*In vitro* plantlets of pear (*P. communis* L. cv. ‘Conference’) were grown on MS medium [40] supplemented with 1 mg L^−1^ 6-BA and 0.2 mg L^−1^ IBA. The plantlets were kept at 25°C with a light intensity of 40 μmol/s/m^2^ and a photoperiod of 16 h/8 h. Subculturing of the *in vitro* plantlets was performed every 3–4 weeks for propagation. Three-week-old plantlets were used for *B. dothidea* infection.

### *Botryosphaeria dothidea* inoculation and stem sample preparation for transcriptome sequencing

The *B. dothidea* strain LW-P [41] was cultured on potato dextrose agar medium at 25°C and fresh growing mycelium was transferred to MS medium under UV light (wavelength = 352 nm) to induce sporulation. The spores were suspended in ddH_2_O at 6 × 10^6^ colony-forming units (cfu)/mL. The middle of the stem of *in vitro* pear plantlets was slightly pricked and inoculated with 1 μL ddH_2_O or spore suspension. The inoculated plants were returned to MS medium and culture was continued at 25°C with 40 μmol/s/m^2^ light intensity and a 16-h/8-h photoperiod. Stem segments (0.5 cm) just above and below the lesion on *B. dothidea*-infected plants and mock-inoculated plants at corresponding positions were cut and collected at 36 and 60 hpi. Stem materials from three plantlets were mixed as one biological sample and each treatment at different time points contained three independent biological replicates.

### RNA extraction and deep sequencing

Total RNA was extracted using the RNAprep Pure Plant Plus Kit (Tiangen, China) and examined for quality and concentration using a Nanodrop spectrophotometer (Thermo, USA). Ribosomal RNA was removed from 2 μg total RNA of each sample using the Ribo-Zero™ rRNA Removal Kit (Epicentre, USA). First-strand cDNA was synthesized using random primers and the RNA strand was degraded by RNaseH. After synthesizing second-strand cDNA with dUTP, the double-stranded cDNA was connected to the sequencing adapter. The library fragments were purified using the AMpure XP system (Beckman Coulter, USA) and enriched using Illumina PCR Primer Cocktail in a 15-cycle PCR reaction. The libraries were then sequenced on the NovaSeq 6000 platform (Illumina) by Personal Biotechnology Co. Ltd (Shanghai, China).

### Sequencing data filtering and genome mapping

The raw data were filtered using Cutadapt software [42] and clean reads were acquired by removing reads containing poly-N and reads containing adapter. The filtered reads were then mapped to the pear genome (http://peargenome.njau.edu.cn) using TopHat software [43].

### Screening and identification of lncRNAs

Transcript assembly was conducted using StringTie [44]. Candidate lncRNAs were screened from assembled transcripts based on information on transcript features and genome location. The protein-coding potential of candidate lncRNAs was analyzed using CNCI [45], PLEK [46], and Pfamscan [47] software. The intersection of non-coding transcripts judged by the three softwares was considered to constitute credible lncRNAs. The identified lncRNAs were categorized based on the relationship with nearest protein-coding genes using FEELnc software [48].

### Gene differential expression and cluster analysis

FPKM values, which were used to indicate expression levels, were calculated using StringTie [44] software. Differential expression analysis was conducted for genes in *B. dothidea*-infected and ddH_2_O mock-inoculated samples using DESeq software [49]. The expression levels of lncRNAs in samples inoculated with *B. dothidea* were compared with those of samples inoculated with ddH_2_O at 36 and 60 hpi, of which the two comparison groups were designated as CK_36 vs Bd_36 and CK_60 vs Bd_60. Genes with *P*-value <0.05 and |log_2_FoldChange| > 1 were considered to be significantly differentially expressed. Clustering analysis was conducted using the pheatmap package in R software [50].

### *Cis*- and *trans*-target gene prediction of lncRNAs

The *cis*- and *trans*- regulated target genes were predicted as previously reported [51]. The protein-coding genes within 100 kb adjacent to the identified lncRNAs were screened as corresponding *cis*-regulated target genes. The *trans*-regulated target genes were predicted by Pearson correlation analysis. The protein-coding genes with Pearson correlation coefficients >0.85 and *P*-value <0.05 were identified as the *trans*-regulated target genes of corresponding lncRNAs.

### GO and KEGG enrichment analysis

GO enrichment analysis of differentially expressed lncRNAs was conducted using topGO software [52] and KEGG enrichment analysis was performed using clusterProfiler software [53].

### Semiquantitative RT–PCR

Semiquantitative RT–PCR was conducted as previously reported [12]. Total RNA extracted from different materials was determined using a Nanodrop spectrophotometer and 1 μg RNA was reverse-transcribed to cDNA using HiScript II Q RT SuperMix with gDNA wiper (Vazyme, Nanjing, China). Semiquantitative PCR was performed using 25–30 cycles. *PcActin* and *Atβ-tubulin2* served as internal housekeeping genes to normalize expression. Primers used in semiquantitative RT–PCR are listed in Supplementary Data Table S9.

### (Stem–loop) RT–qPCR

RT–qPCR was performed as previously reported [54]. *PcActin* served as reference gene for analysis of expression levels of mRNA. *PcU6* served as reference gene for analysis of expression levels of miR399b. Stem–loop RT–qPCR was performed as previously reported [54] to detected miR399b. Each treatment contained three biological replicates and was analyzed by the 2^−ΔΔCt^ method. Primers used in RT–qPCR are listed in Supplementary Data Table S9.

### Prediction of endogenous target mimics

The sequences of mature miRNAs in pear were obtained by small RNA sequencing which was described in a previous report [21]. The identified lncRNAs were analyzed with miRNAs in pear using the online tool RNAhybrid (https://bibiserv.cebitec.uni-bielefeld.de/rnahybrid). The screening rules were as previously reported [12] according to the sequence characteristics of eTMs.

### Transient co-transformation in tobacco leaves to verify regulation by MSTRG.32189 of miR399b-*PcUBC24*

The regulatory effect of MSTRG.32189 on miR399b-*PcUBC24* was examined by transient co-transformation in tobacco leaves. MSTRG.32189 and PcmiR399b were cloned using cDNA from *P. communis* L. cv. ‘Conference’ plantlets and constructed into pBI121 to generate 35S:MSTRG.32189 and 35S:PcmiR399b. Mutant MSTRG.32189 with four bases mutated at predicted target sites was selected as previously reported [12] and was obtained by overlap extension PCR and cloned into pBI121 to generate 35S:mMSTRG.32189. The sequence of the *PcUBC24* target site was amplified and fused to the N-terminus of GFP in the pMS4 vector. The primers are listed in Supplementary Data Table S9. Each constructed plasmid was transformed into GV3101. *Agrobacterium* cells with different combinations of constructs (35S:*PcUBC24*ts-*GFP*, 35S:*PcUBC24*ts-*GFP* + 35S:PcmiR399b, 35S:*PcUBC24*ts-*GFP* + 35S:PcmiR399b + 35S:MSTRG.32189, 35S:*PcUBC24*ts-*GFP* + 35S:PcmiR399b + 35S:mMSTRG.32189) were diluted in the same proportions and infiltrated into tobacco leaves. Fluorescence signals of GFP were observed at 48 hpi under UV light. Meanwhile, GFP mRNA expression levels were analyzed by RT–qPCR using three independent biological replicates. For the validation of the cleavage site of miR399b on the target gene *PcUBC24*, leaf tissues co-expressing miR399b and the *PcUBC24* target site were taken, and RNA was extracted for RACE assay as previously reported [54].

### Genetic transformation of *Arabidopsis* and *B. cinerea* infection assay

For MSTRG.32189 overexpression, MSTRG.32189 was amplified and cloned into pBWA vector to generate the 35S:MSTRG.3218 construct. The expression vectors were transformed into GV3101 and *Arabidopsis* Col-0 seedlings were transformed using the floral dip method [55]. Transgenic plants were selected using 15 mg/L BASTA and identified by PCR. Expression levels of MSTRG.32189 and target genes were analyzed by semiquantitative RT–PCR. The *T*_3_ seedlings of the transgenic plants were grown at the same time under identical conditions for disease resistance and Pi content analysis. The *B. cinerea* strain B05.10 provided by Prof. Guoqing Li from Huazhong agricultural university in China [56] was used to infect *Arabidopsis* seedlings. After ~2 weeks of cultivation at 25°C in PDA medium, conidia were produced and the spore suspension was prepared using PDB medium and filtered using sterile filter paper. The spore suspension at the concentration of 5 × 10^5^ cfu/mL was inoculated into 4-week-old *Arabidopsis* plants with consistent growth status. Three independent experiments were conducted and five plants per line, with three to five leaves per plant, were inoculated each time. The inoculated *Arabidopsis* plants were placed in a transparent culture box with high relative humidity. Three days after inoculation the diameter of the lesion was measured. The primer sequences are listed in Supplementary Data Table S9

### Plasmid construction for overexpression and knockout of genes

The overexpression plasmids MSTRG.32189 and PcmiR399b were constructed into pBI121 as previously reported [54]. The amplified MSTRG.32189 full-length and PcmiR399b precursor sequences were ligated into the pBI121 vector between endonuclease sites XbaI and SacI.

The overexpression plasmid *PcUBC24* was constructed into pK7WG2D vector following a previous report [57]. The product of gene-specific primer amplification was used as a template for amplification with attB1 and attB2 universal primers. The purified product was mixed with the pDONR221 vector for the BP reaction and the positive clone was then sequenced correctly. After the LR reaction, *PcUBC24* was ligated to the PK7WG2D plasmid, and after sequencing and analysis of the positive clones the PK7WG2D-eGFP-*PcUBC24* recombinant plasmid was obtained.

TCRISPR/Cas9 plasmids for genome editing of the *PcUBC24* gene were constructed following a previous report [58]. The target sites for *PcUBC24* were selected using the web program CRISPR-P (http://cbi.hzau.edu.cn/crispr/) [59] and the primer sequences for generating corresponding guide RNAs are listed in Supplementary Data Table S9. The sgRNA cassettes containing target sequences were generated by overlapping PCR and constructed into the *pKSE401* binary vector (Supplementary Data Fig. S17; https://www.addgene.org/62202/) using the Golden Gate assembly method [60]. The PKSE401-Cas9-*PcUBC24* construct was transformed into *Agrobacterium* strain EHA105 for pear callus transformation.

### Genetic transformation of pear calluses and *B. dothidea* inoculation

The method of pear callus transformation was as previously reported [61] with slight modifications. Briefly, MSTRG.32189 and PcmiR399b were constructed into pBI121 and the recombinant plasmids were transformed into *Agrobacterium* strain EHA105. Pear calluses were immersed in *Agrobacterium* suspension for 20 min, blotted dry on sterile filter paper and then transferred to solid MS basal medium for 2 days in darkness at 24°C. Next, calluses were transferred to MS basal medium supplemented with 50 mg/L kanamycin and 200 mg/L timentin. Transformed calluses were selected and subcultured before analysis. Positive transgenic calli were examined by PCR using DNA as template and RT–qPCR using cDNA as template. Target gene fragments in knockout-*PcUBC24* callus were further amplified and 30 clones of each line were randomly selected for Sanger sequencing to evaluate the editing efficiency and mutation type.

Transgenic and wild-type calluses were then challenged with 2 μL *B. dothidea* spore suspension at a concentration of 3 × 10^6^ cfu/mL. The transgenic and WT calluses were laid flat on MS medium and spore suspension was added to the culture at 25°C. The diameter of the lesion was determined at 60, 80, 100, and 120 h after inoculation.

### Determination of Pi content

Pi content determination was performed as previously described [62]. Briefly, plant materials were ground to fine powder and suspended in 1% glacial acetic acid. After centrifugation at 8000 g for 15 min, 500 μL supernatant was added to to 700 μL Pi content determination buffer (mixture of 10% ascorbic acid and 0.42% ammonium molybdate·4H_2_O at 6:1 ratio) and incubated in a water bath at 45°C for 20 min. Two hundred microliters of reaction liquid was used to measure absorbance at 820 nm and Pi content was calculated relative to fresh weight.

### ROS level determination

ROS determination was conducted by using a luminol-based assay as previously reported with little modification [24]. Each sample was weighed and 0.3 g of sample was treated with 1 μL of lumefantrine and horseradish peroxidase and 2 μL of 50 μg/mL chitosan in 100 μL solution for 10 h at 76 rpm in the dark. Chemiluminescence was detected after treatment with a SPARK microplate reader (Tecan, Switzerland).

### Statistical analysis

Results are expressed as mean ± standard deviation of at least three biological replicates. Significance differences were determined by Student’s *t*-test.

## Supplementary Material

Web_Material_uhae359

## Data Availability

Sequence data generated during this work can be found in the GenBank database. Sequences of MSTRG.32189 and *PcUBC24* CDS have been uploaded to GenBank under accession numbers PP104288 and PP104289, respectively. RNA-seq raw reads used for pear lncRNA analysis in this study have been uploaded to NCBI under accession number PRJNA1072809.
