# Supplementary material for: The long non-coding RNA MSTRG.32189-PcmiR399b-PcUBC24 module regulates phosphate accumulation and disease resistance to Botryosphaeria dothidea in pear
Source: Hortic Res. 2025 Jan 3;12(4):uhae359. doi: 10.1093/hr/uhae359 (PMC11891480; doi:10.1093/hr/uhae359)
Supplement: Web_Material_uhae359 [file web_material_uhae359.zip › Supplemental figures - revised 12.docx]

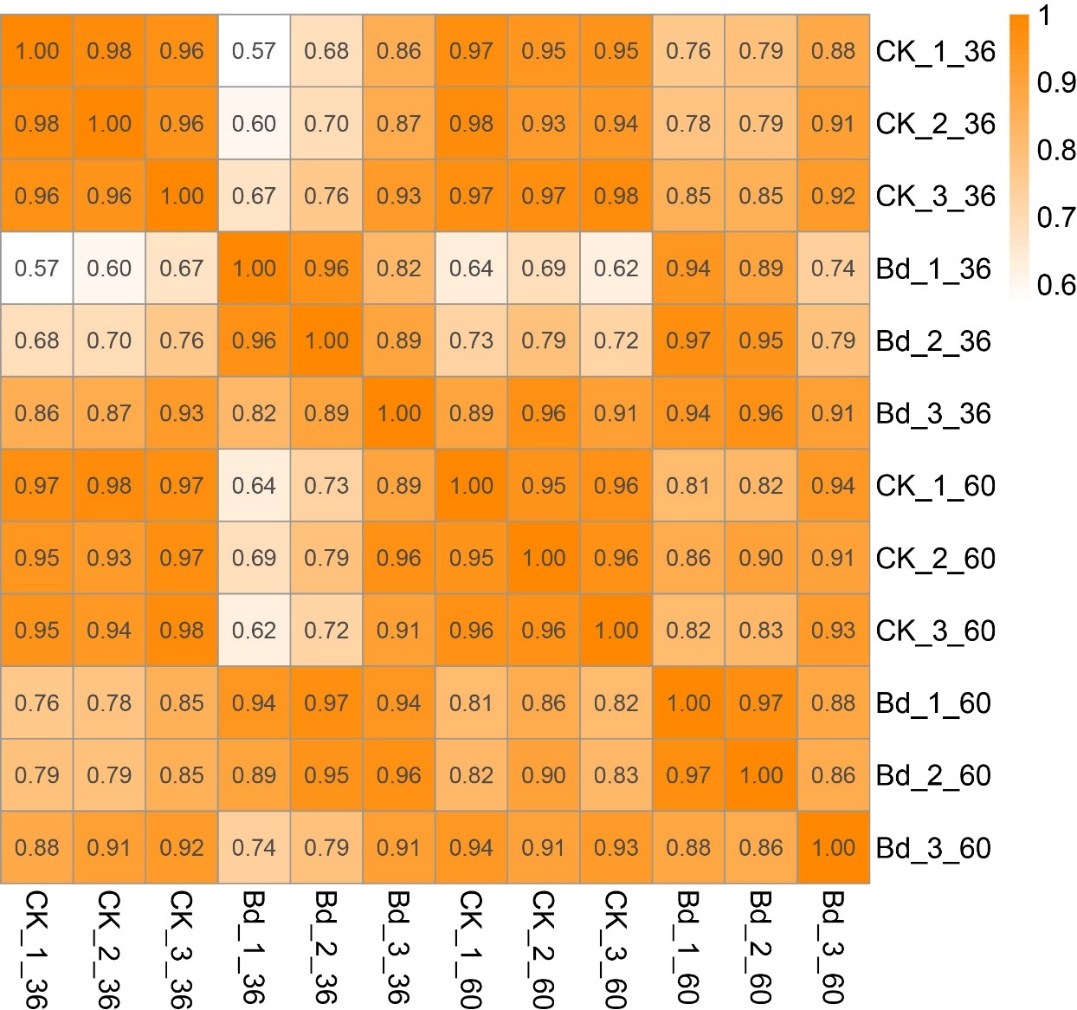


**Fig. S1 The Pearson correlation analysis among 12 libraries.** Numbers in each grid indicates Pearson correlation coefficients of different group.


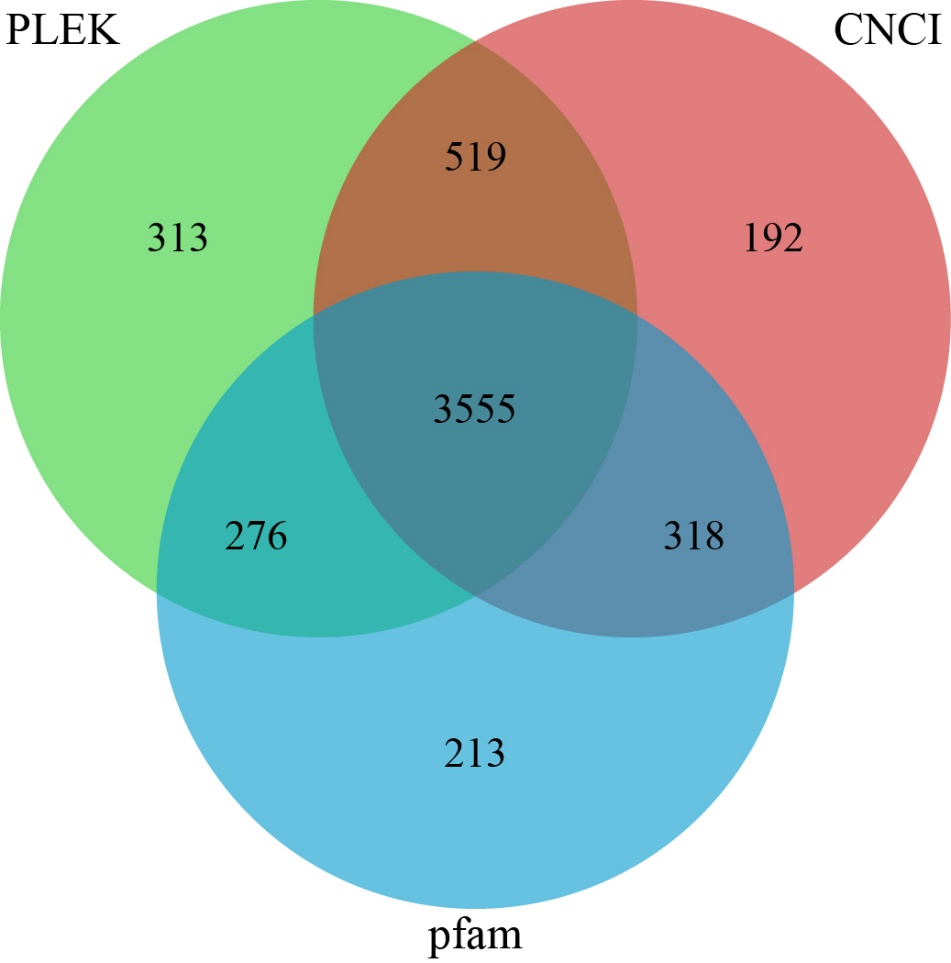


**Fig. S2 The protein-coding potential analysis of candidate lncRNAs using software of CNCI, PLEK and Pfamscan respectively.**


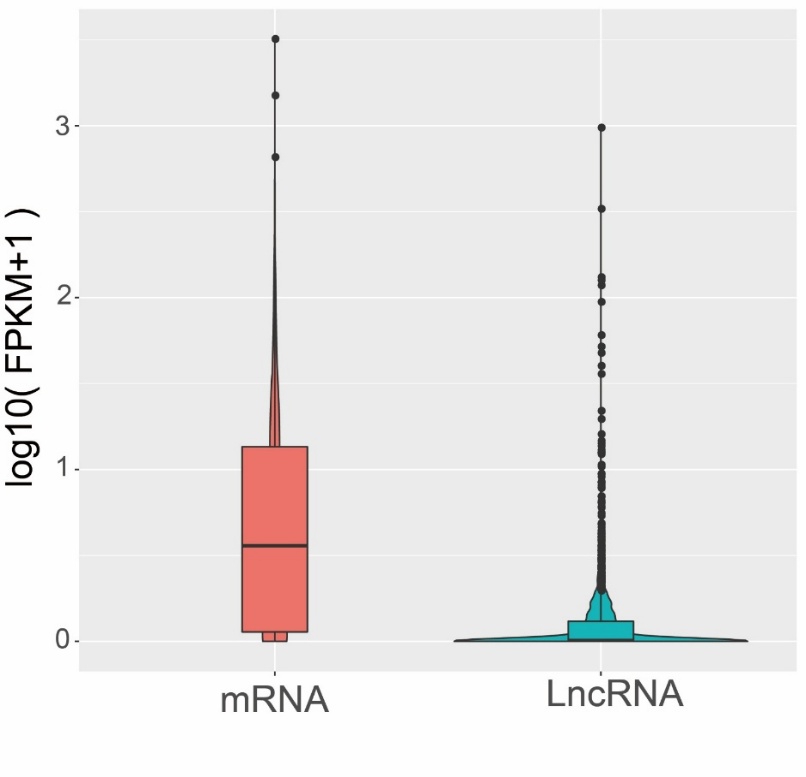


**Fig. S3 Comparison of expression abundance between mRNAs and lncRNAs.**

**Fig. S4 Characterization analysis of lncNATs.** A, KEGG enrichment of protein-coding genes target by lncNATs (resistance related pathways were marked with red triangles); B, Co-expression correlation analysis of lncNAT-mRNA pairs; C, Heatmap analysis showing the expression pattern of negative-regulated lncNAT-mRNA gene pairs

**Fig. S5 GO enrichment of trans-regulated genes in CK_36 vs Bd_36 (a) or CK_60 vs Bd_60 (b).**

**Fig. S6 Expression level verification of 4 pairs of predicted cis/trans-function lncRNAs/mRNA genes.** FPKM values of cis- (a, b) and trans-function (c, d) lncRNAs/mRNA genes; Semi-quantitative RT-PCR of cis- (e, f) trans-function (g, h) lncRNAs/mRNA genes.

**Fig. S7 Target sites of predicted Endogenous Target Mimics and corresponding miRNAs.**

**Fig. S8 Expression profiles verification of MSTRG.32189 in response to *Diaporthe eres* (a) and *Valsa pyri* (b) infection at 36 hpi and 60 hpi, and expression profiles of MSTRG.32189 in leaf and stem tissues of 4 pear species (c)** **by semi-quantitative RT-PCR.**

**Fig. S9 Growth phenotype of 4-week-old OE-MSTRG.32189 Arabidopsis**

**Fig. S10 Analysis of the conserved domains of PcUBC24 in pear and AtPHO2 in Arabidopsis.**

**Fig. S11 Identification of the OE-MSTRG.32189 (a) and OE-PcmiR399b (b) transgenic pear callus lines using DNA as template. c, Semi-quantitative RT-PCR of MSTRG.32189 in OE-MSTRG.32189 callus.**

**Fig. S12 Determination of the expression of disease resistance pathway-related genes in OE-MSTRG.32189 and WT pear callus**

**Fig. S13 Identification and Pi content determination of STTM-miR399b transgenic pear callus.**

a, PCR identification of STTM-miR399b pear callus using DNA as template; b, Detection of relative expression level of PcmiR399b in transgenic lines by RT-qPCR. c, Pi content determination of STTM-miR399b transgenic pear callus.

**Fig. S14 Determination of the phosphorus homeostasis-regulated gene expressions in STTM-miR399b and WT pear callus.**

**Fig. S15 Identification of the OE-PcUBC24 and KO-PcUBC24 transgenic pear callus. GFP fluorescence detection of OE-PcUBC24 (a) and KO-UBC24 (c) positive callus under UV light. PCR detection of OE-PcUBC24 (b) and KO-PcUBC24 (d) constructs using DNA as template.**

**Fig. S16 RT-qPCR analysis of *PcUBC24* in transgenic callus of OE-PcUBC24 and WT callus.**

**Fig. S17 Map of pKSE401-CRISPR/Cas9 vector.** In pKSE401-CRISPR/Cas9-PcUBC24-GFP vector construction, replace T1/T2 of intermediate vector in above picture with the target sequences of *PcUBC24*.

**Fig. S18 Analysis of mutation types in transgenic KO-*PcUBC24* callus lines (PAM sequences are bolded in blue; target site sequences are marked in horizontal lines; insertion bases are marked in red; - are deletion bases; and black boxes are termination codons that appeared prematurely due to shifting codes; The ratio of the clones for different mutation type to the total clones is shown on the right)**

**Fig. S19 Disease resistance determination of pear fruits transiently overexpressing *PcUBC24*.** a, Relative expression level of *PcUBC24* in transformed fruits; b, Photographs of representative fruits with median lesion diameter in EV and OE-*PcUBC24*; c, Determination of lesion diameter for different transformants inoculated with *B. dothidea* at 72 hpi.
