# Supplementary figures and images for: The long non-coding RNA MSTRG.32189-PcmiR399b-PcUBC24 module regulates phosphate accumulation and disease resistance to Botryosphaeria dothidea in pear
Source: Hortic Res. 2025 Jan 3;12(4):uhae359. doi: 10.1093/hr/uhae359 (PMC11891480; doi:10.1093/hr/uhae359)

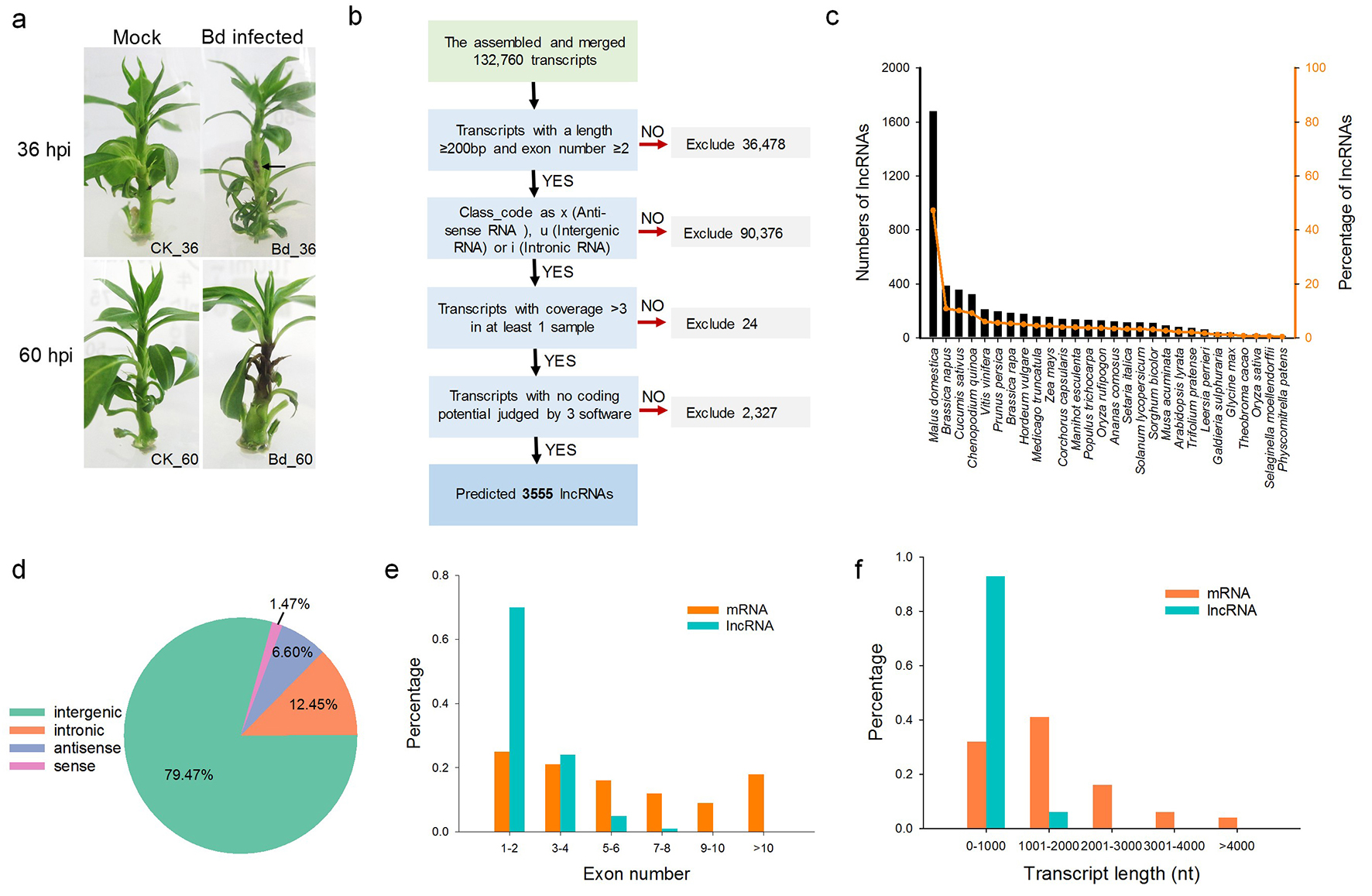

Supplement: Web_Material_uhae359 [file web_material_uhae359.zip › Fig 1.jpg]

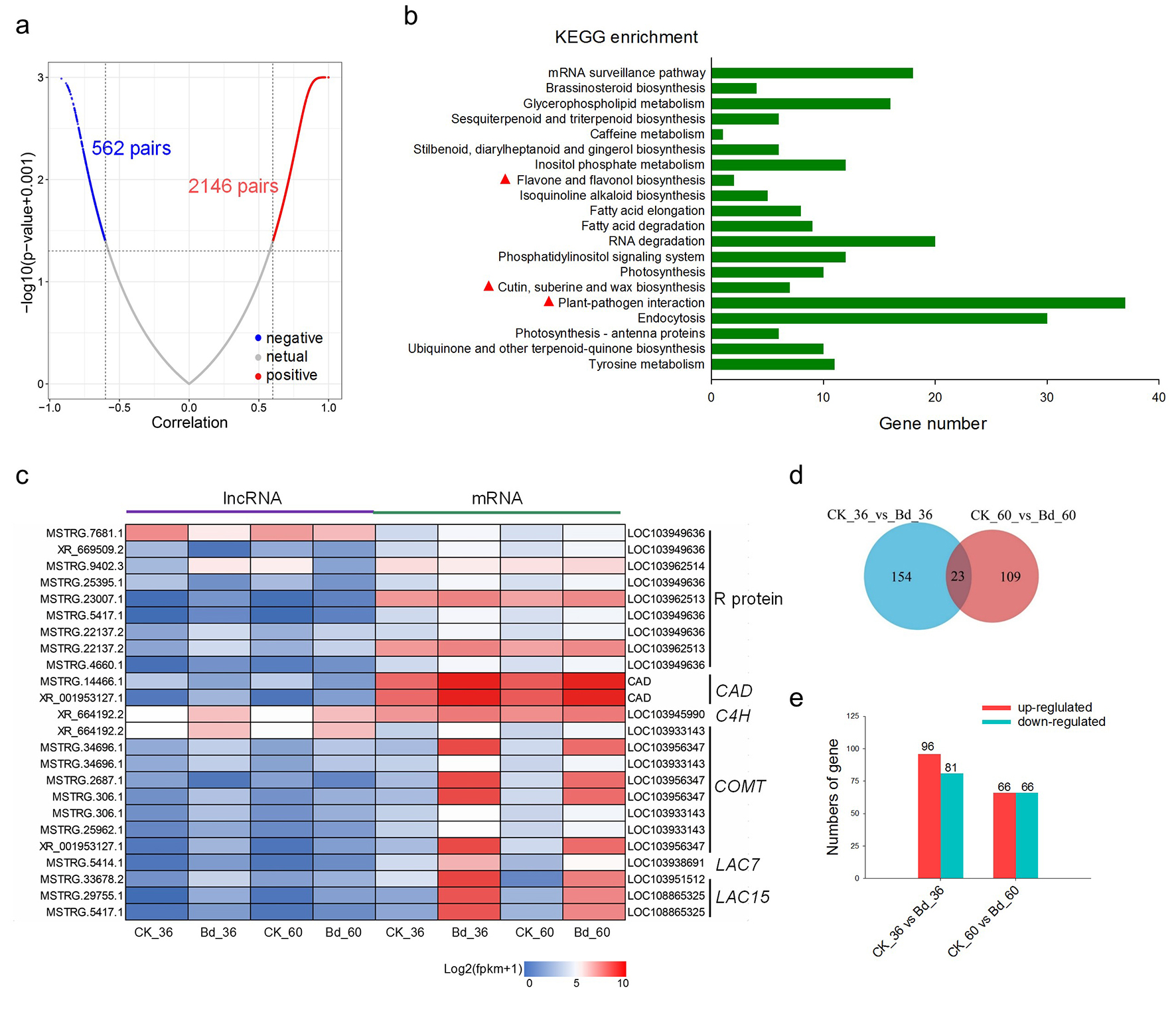

Supplement: Web_Material_uhae359 [file web_material_uhae359.zip › Fig 2.jpg]

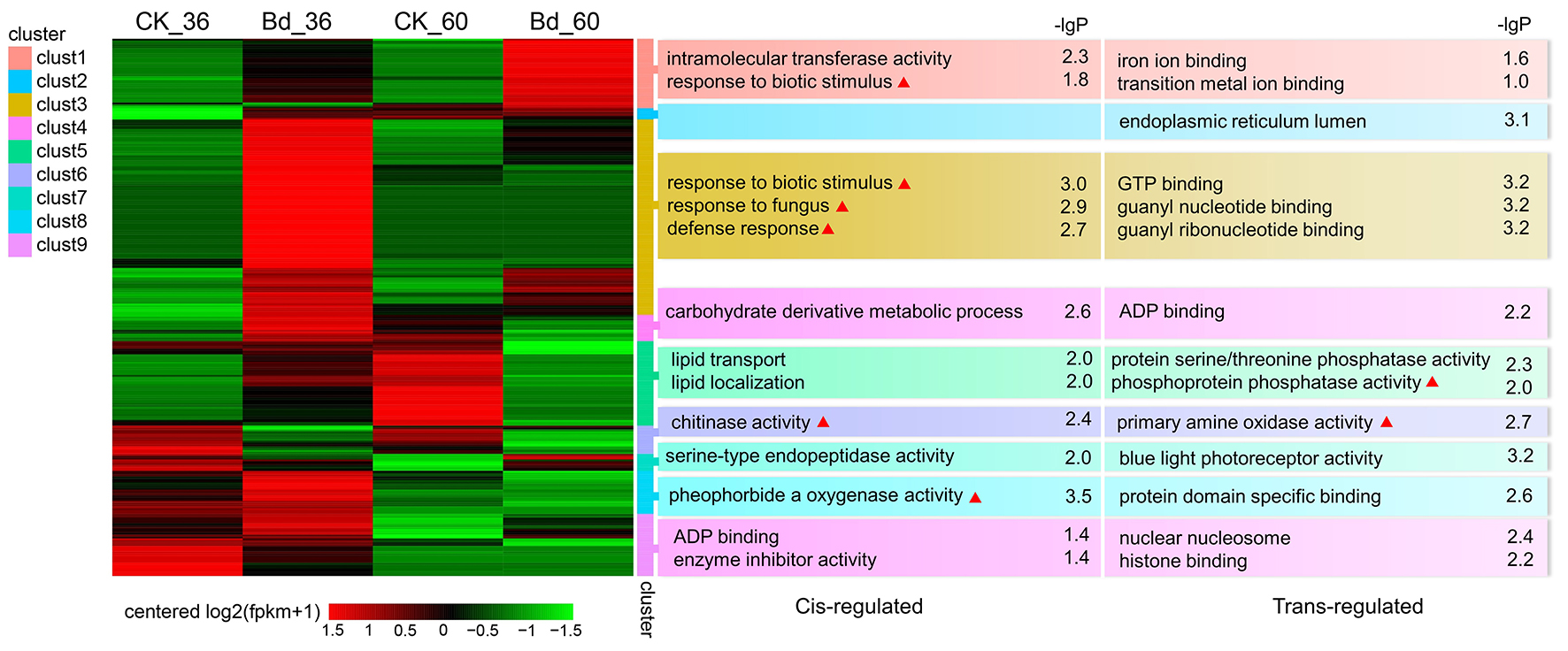

Supplement: Web_Material_uhae359 [file web_material_uhae359.zip › Fig 3.jpg]

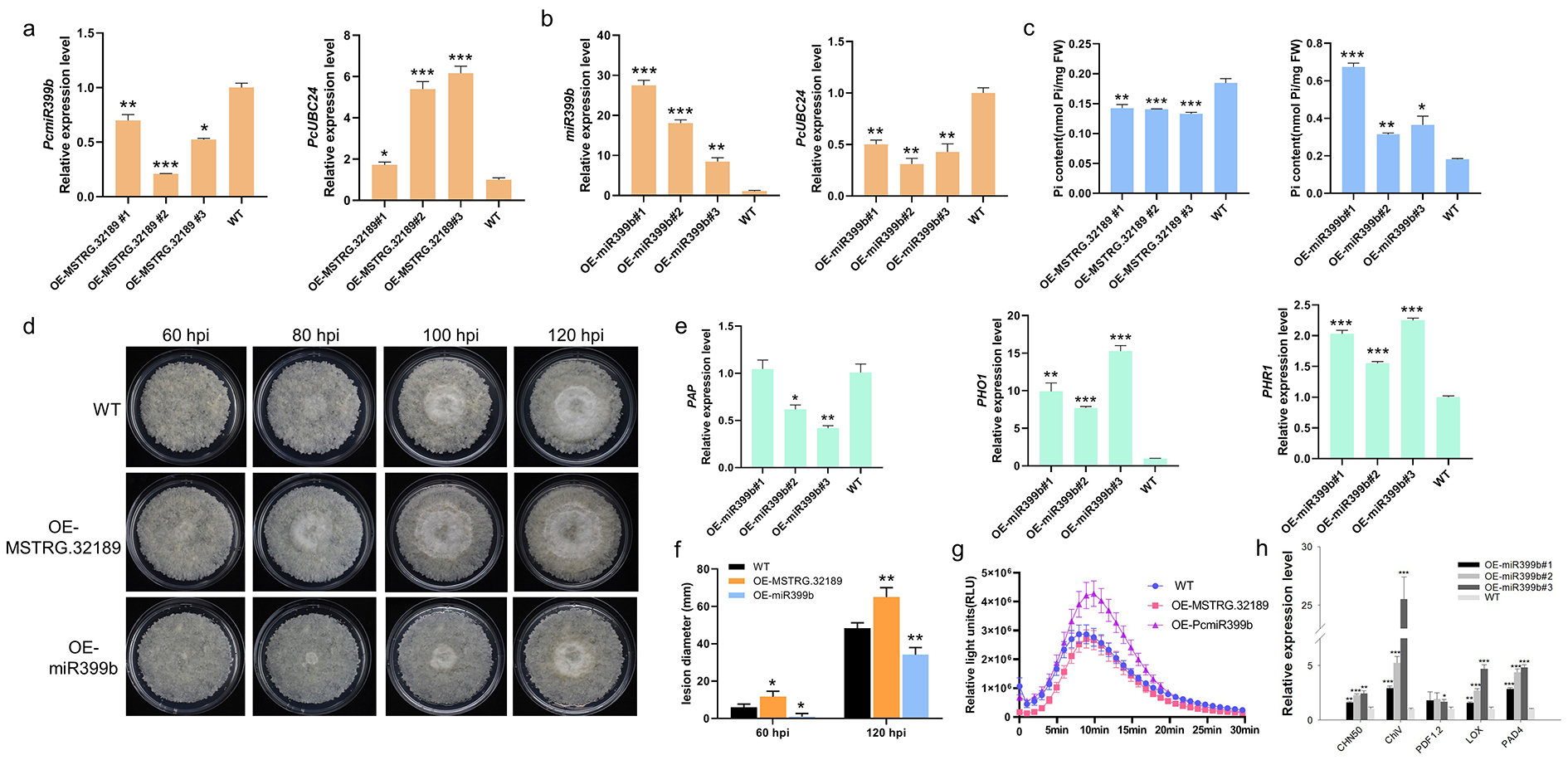

Supplement: Web_Material_uhae359 [file web_material_uhae359.zip › Fig 6.jpg]
